# Gestational Age-Specific Reference Ranges for Fetal Cavum Septum Pellucidum Width in an Iranian Population: Gestational Age-Specific Reference Ranges for Fetal Cavum Septum Pellucidum Width

**DOI:** 10.31661/gmj.v14i.3952

**Published:** 2025-07-25

**Authors:** Elaheh Zarean, Somayeh Khanjani, Farinaz Farahbod, Atefeh Batebi

**Affiliations:** ^1^ Department of Perinatology, Faculty of Medicine, Isfahan University of Medical Sciences, Isfahan, Iran

**Keywords:** Fetus, Cavum Septum Pellucidum, Ultrasonography, Prenatal, Gestational Age, Reference Values

## Abstract

**Background:**

Cavum septum pellucidum (CSP) is a critical anatomical land mark of normal midline development of brain and its absent is associated with brain anomalies. On the other hand, the size of CSP differs among different populations. The aim of this study was the evaluation of the size (width) of CSP at various gestation age (18 to 40 weeks’ pregnancy) in Isfahan, Iran population.

**Materials and Methods:**

This cross-sectional study was performed at Isfahan hospitals (Al-Zahra and Shahid Beheshti) from December 2022 until December 2023. The CSP was measured in 1000 normal fetuses at trans-ventricular plane by trans-abdominal ultrasound by two expert operators. In these fetuses the HC and BPD were measured. Regression analysis revealed significant relationship between CSP width and gestational age, also between CSP width and HC and BPD.

**Results:**

The mean CSP width was 5.3 ± 2.08 mm, ranging from 2.15 mm to 8.85 mm. CSP width increased progressively from 18 to 33 weeks, plateaued at 33–37 weeks, and slightly declined near term. Strong correlations were found between CSP width and head circumference (HC) (r=0.644, P0.01) and biparietal diameter (BPD) (r=0.631, P0.01). The study defined normative fetal CSP development values using percentile distributions with a 5th percentile length-to-width ratio of 1.06.

**Conclusion:**

The present study provides normative data for fetal CSP width and useful information about the development of the CSP based on gestational age.

## Introduction

The visualization of CSP is considered an integral part of the prenatal second and third trimester sonographic evaluation of the fetal neural axis [1][2]. Also, it is critical anatomical land mark of the normal midline development of fetus’s brain [3]. The septa pellucida are two, thin translucent leaves that extend from the anterior part of the body, the genu and the rostrum of the corpus callosum to the superior surface of fornix. Fetuses begin to develop at 10-12 weeks of gestation and reach an adult form by the 17th week of gestation [4]. It should always be visible between 18-37 weeks or when the biparietal diameter measures 44-88 mm. Failure to visualize it after 37 weeks is almost certainly due to the normal obliteration if brain is otherwise normal [5][6]. Failure to demonstrate the CSP with antenatal ultrasound is typical of many cerebral anomalies, such as holoprosencephaly, schizencephaly, agenesis of the corpus callosum and septo-optic dysplasia [7][8][9][10][11]. This study is novel and clinically significant because, until now, Iranian physicians have relied on CSP reference ranges derived from European or American populations, which may not accurately reflect normal fetal brain development in Iranians due to potential ethnic, genetic, or anthropometric differences. Using foreign standards risks misdiagnosis, either overestimating abnormalities in healthy fetuses or missing true anomalies. By establishing the first gestational age-specific reference ranges for CSP width in an Iranian population, this study provides locally applicable normative data, ensuring more accurate prenatal assessments. Additionally, since Iran’s legal abortion cutoff is 18 weeks and 5 days, precise CSP measurements are crucial for timely decisions and borderline values based on Western data could lead to unnecessary terminations or missed interventions.

## Materials and Methods

**Figure-1:**
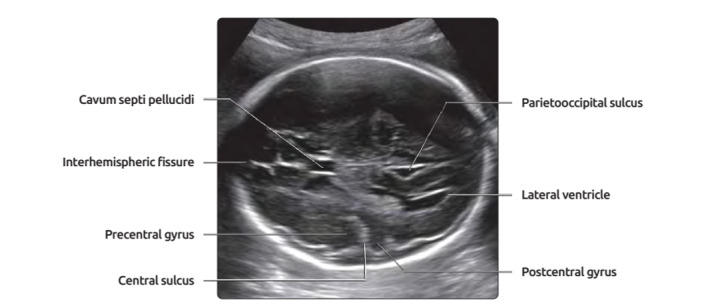
Figure 1. Trans-ventricular view [3].

This was a cross-sectional study that CSP width of 1000 pregnancies with a gestational age of 18 and 40 weeks were evaluated retrospectively. Eligible patients who were referred to Al-Zahra and Shahid Beheshti hospital of Isfahan, Iran from December 2022 until December 2023. The Isfahan Center for Obstetrics is one of the leading referral centers in Iran, attracting patients from various cities across the country (Shahrekord, Bandarabbas, Ahvaz, Bushehr, and etc.). Due to its high reputation and specialized services, the center receives a broad spectrum of patients from different geographical, socioeconomic, and cultural backgrounds. This wide referral range ensures that the sample is not limited to a specific subgroup. Gestational age was confirmed in all cases either by last menstrual period and or an ultrasound scan that was performed in the first trimester.

All ultrasound were performed by two operators (one operator did the measurements and the next operator checked them to ensure validity of measurements) with one of the three ultrasound devices (Philips Affinity 70, Philips Healthcare, Netherlands; Philips Affinity 50Philips Healthcare, Netherlands; GE Voluson E6, General Electric, USA) by using a 2-9 MHz transducer and transabdominal method.

All fetuses had normal brain scan in examination, CSP was identified on axial trans-ventricular plane and antero-posterior diameter on the inner borders of its proximal and distal bounding (inner to inner) and the length of CSP (inner to inner). The BPD and HC were measured in trans-thalamic plane in these fetuses. All these results were recorded in the information list. A polynomial regression model was used to assess the relationship between CSP width and BPD, CSP width and HC. No follow-up was considered as this study was conducted retrospectively.

### Outcome Measures

The primary outcome measure of this study was the antero-posterior diameter of the CSP, measured in millimeters. Secondary outcomes included head circumference (HC) and biparietal diameter (BPD), both assessed to evaluate their correlation with CSP width.

### Statistical Analyses

Statistical analyses were performed using polynomial regression models to examine the relationship between CSP width and both HC and BPD. Pearson’s correlation coefficient (r) was used to determine the strength and significance of these associations. Descriptive statistics, including mean ± standard deviation (SD) and 2SD ranges, were computed for CSP width, HC, and BPD across gestational ages. Percentile categorizations (5th-90th) were derived to establish normative reference ranges for CSP width. The CSP length-to-width ratio was also analyzed by percentile, with the 5th percentile ratio being 1.06. All analyses were conducted using SPSS software (version 21), with significance set at P<0.05.

## Results

**Table:** Table1. The Mean Size (mm) of HC, BPP and CSP basaed on GA

GA (week)	Number	HC (mean±SD)	BPP (mean±SD)		CSP diameter	
				-2SD	Mean	+2SD
19 (18-19+6d)	80	156.97±3.4	41.93±0.43	2.15	3.22	4.29
21 (20-21+6d)	88	177.83±2.14	46.89±0.68	2.51	4.01	5.5
23 (22-23+6d)	72	191.62±11.1	56.38±0.95	3.38	4.58	5.78
25 (24-25+6d)	69	259.43±31.65	61.01±0.49	3.7	5.09	7.29
27 (26-27+6d)	66	244.45±3.53	65.28±1.17	3.65	5.49	7.33
29 (28-29+6d)	115	285.27±21.86	71.25±0.71	3.62	5.63	7.64
31 (30-31+6d)	124	278.9±1.7	81.94±3.01	3.87	5.87	7.87
33 (32-33+6d)	112	300.28±1.6	83.5471±1.35	3.84	6.23	8.62
35 (34-35+6d)	113	308.19±2.24	87.95±1.58	3.93	6.15	8.37
37 (36-37+6d)	116	318.3±1.76	88.46±0.41	3.66	6.24	8.79
39 (38-40)	45	315.66±4.99	88.13±1.42	3.94	6.08	8.85

**Figure-2:**
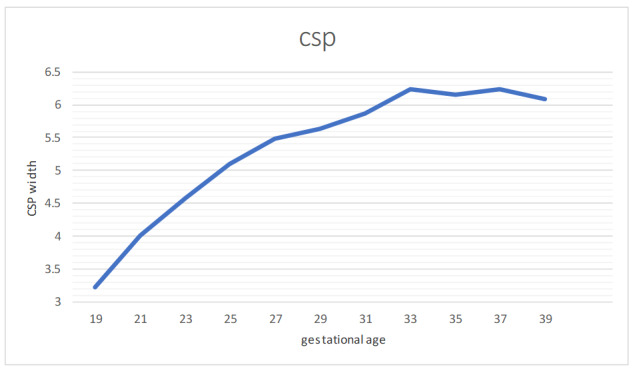
Figure 2. CSP diamter (mm) based on GA

**Figure-3:**
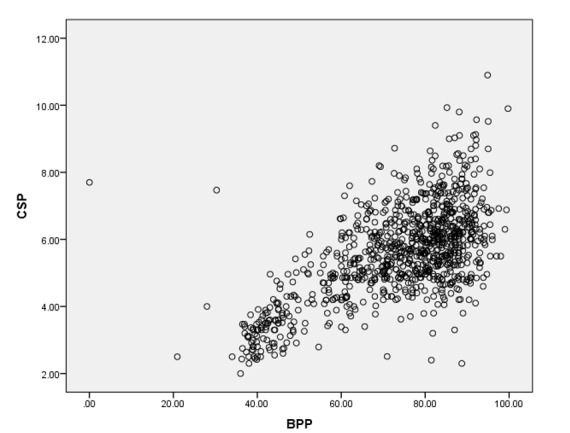
Figure 3. Correlation between CSP and BPP

**Figure-4:**
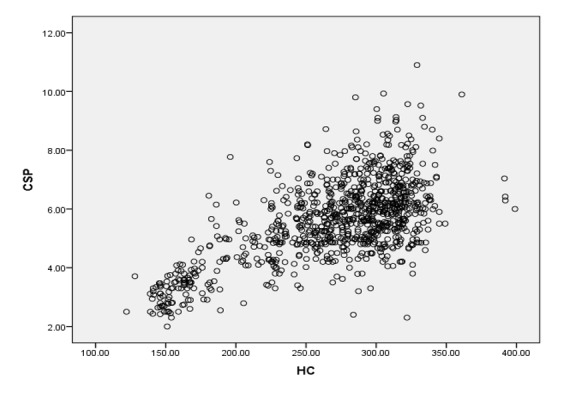
Figure 4. Correlation between CSP and HC

In total, we evaluated 1000 consecutive uncomplicated singleton pregnancies between 18 and 40 weeks of gestation.

Table-1 presents the mean size (mm) of head circumference (HC), biparietal diameter (BPP), and cavum septi pellucidi (CSP) diameter across different gestational ages (GA) in weeks, ranging from 19 to 39 weeks. The data includes the number of observations per GA group, along with mean ± standard deviation (SD) values for HC and BPP, while CSP diameter is detailed with mean values and ±2SD ranges. HC and BPP measurements show a progressive increase with advancing GA, with HC rising from 156.97±3.4 mm at 19 weeks to 318.3±1.76 mm at 37 weeks, and BPP increasing from 41.93±0.43 mm to 88.46±0.41 mm in the same period. CSP diameter also grows with GA, with mean values increasing from 3.22 mm at 19 weeks to 6.24 mm at 37 weeks, though with some variability in the ±2SD ranges, as shown in Figure-2.

**Table:** Table2. Percentile Categorization of the CSP in Study Population

Gestational week	5	10	25	50	75	90
18-20	2.4475	2.5	2.7625	3.325	3.6075	4.005
20-22	2.694	2.918	3.47	3.97	4.76	5.468
22-24	3.6695	3.947	4.2425	4.59	5.175	5.877
24-26	3.342	3.633	4.1975	5.015	5.75	6.6631
26-28	4.1315	4.57	4.86	5.44	6.1	6.603
28-30	4.083	4.409	4.8975	5.695	6.315	7
30-32	4.291	4.5	5.085	5.83	6.51	7.218
32-34	4.4605	4.95	5.5	6.235	7.07	7.597
34-36	4.5105	4.794	5.3375	6.04	6.7175	7.586
36-38	4.54	4.8	5.5	6.17	6.92	8.124
38-40	4.48	4.746	5.2475	5.705	6.2225	7.426

Pearson correlation test revealed significant statistical correlation between CSP width and HC (r=0.644, P-value<0.01), and CSP width and BPP (r=0.631, P-value<0.01), as shown in Figure -3 and -4. The percentiles (5th, 10th, 25th, 50th, 75th, 90th) show a general increase in CSP width with advancing GA, though with some fluctuations. For example, the median (50th percentile) CSP width grows from 3.325 mm at 18-20 weeks to 6.17 mm at 36-38 weeks, before slightly decreasing to 5.705 mm at 38-40 weeks. Variability is evident in higher percentiles, with the 90th percentile peaking at 8.124 mm (36-38 weeks). Table-3 presents the CSP length-to-width ratio across different percentiles (5th, 25th, 50th, 75th, 90th, 95th), showing a progressive increase. The median ratio is 1.53, rising to 2.0 at the 90th percentile and 2.2 at the 95th percentile, indicating that CSP morphology becomes more elongated in higher percentiles.

**Table:** Table3. CSP length to width ratio based on percentile catogorazation

	5	25	50	75	90	95
Ratio	1.06	1.14	1.53	1.81	2	2.2

## Discussion

Together, our study provides reference values for assessing fetal brain development, with CSP metrics particularly useful for detecting potential anomalies in midline brain structures. Falco et al. (in Italy) have suggested that CSP is frequently impossible to visualize prior to 18 weeks of gestation or with a BPD of less than 44 mm. If using transabdominal sonography between 18 and 37 weeks or with a BPD 44 and 88 mm the CSP is always seen. The mean width of the CSP was 5.3±1.7 mm (rang 2-9 mm). The CSP was found to increase with gestational age and BPD diameter, with a slight decrease around term [12]. In another study Rakic and Yakovlev's have suggested that between 19 and 42 weeks of gestation the CSP was identical in all cases with exception of one fetus of 38 weeks. The CSP width was found increase with gestational age of 19 to 27 weeks and to plateau between 27 weeks and term [13]. Jon et al. also showed that CSP increased gradually between 19 and 27 weeks of gestation and then plateaued between 28 and term. Regression analysis revealed significant association between CSP width and gestational age and CSP width and BPD. In pervious study the range of CSP width was from 2-10 mm [4]. Our study showed that CSP width increased gradually from 18 to 33 weeks of gestation and then plateaued between 33 and 37 weeks with slight reduction around term. The range of CSP width was from 2.15 and 8.85 mm. The mean width of CSP was 5.3±2.08 mm. Arisoy et al. conducted the mean between 15-26 weeks of gestation CSP width was found to be 4.1±0.8 (1.6-7.7mm) and significantly positively with gestational age, BPD and HC. CSP 50th percentile values were found to be 2.8-5.9 mm at 15-28 [14]. In their perspective study Serhatlioglu et al. reported that CSP width was between 0.6 and 9 mm at 16-38 weeks of gestation and increased significantly with gestational age and BPD [15].

Our study revealed moderate statistical correlation between CSP width and BPD also CSP with and HC. Tao et al. evaluated CSP width in 322 singleton pregnant women between 25 and 39 weeks of gestation and found the mean CSP width to be 6.36±1.2 mm (range 3.4-10mm) and reported that there was no significant correlation between CSP width and gestational age. This finding differs from our study [16]. Similar study was performed in Iranian population at Zahedan. The mean cavum septum pellucidum size in the second trimester was 3.71 ± 0.81 and 6.11 ± 1.09 mm in the third trimester. The mean size of cavum septum pellucidum in the second trimester of pregnancy was significantly lower than in the third trimester. A total of 500 fetuses were evaluated in this study [17]. The corpus callosum is closely related anatomically and embryologically to the CSP. Most authors state that there cannot be a CSP without a corpus callosum. Both the CC and CSP develop from commissural plate. The diagnosis of fetal complete agenesis of the CC is often based on the absence of the CSP, additional finding includes tear drop-shape lateral ventricle. Partial agenesis of CC (PACC) remains a challenge. Karl et al. devise a method to measure the area of the CSP and the length to width ratio (CSP ratio). In normal population, the length and width of the CSP increases with increasing BPD. The CSP ratio was small (<5th percentile) in 95% (19/20) of partial agenesis of CC and empirical cut-off of <1.5. 80% of PACC fetuses were below this threshold. Analysis of Z-score showed that fetuses with PACC had a significantly smaller CSP ratio [18]. In our study all fetuses had normal brain scan. The CSP ratio for 5th percentile was 1.06.

## Conclusion

In summary, our findings suggest that between 18 and 40 weeks of gestation, a conventional transabdominal ultrasonography should always be performed to visualise the CSP in healthy foetuses. The CSP diameter rises steadily before plateauing. Additionally, our results showed a moderate statistical coorelation between HC and CSP and CSP width . This study provides Iranian-specific reference ranges for CSP measurements, allowing physicians to more accurately assess fetal brain development in the local population and reduce diagnostic errors caused by applying foreign standards. By adopting these data, clinicians can improve prenatal care by minimizing false positives and ensuring true anomalies are correctly identified based on ethnically relevant norms.

## Conflict of Interest

None.
